# Effect of group-based acceptance and commitment therapy on older stroke survivors: study protocol for a randomized controlled trial

**DOI:** 10.1186/s12906-023-04160-z

**Published:** 2023-10-06

**Authors:** Furong Chen, Qiao Zhou, Junqi Wu, Xianghua Xu

**Affiliations:** 1 The Affiliated Cancer Hospital of Xiangya School of Medicine, Central South University/Hunan Cancer Hospital, Changsha City, Hunan Province 410013 China; 2 School of Nursing, University of South China, Hengyang City, Hunan Province 421001 China; 3 The Third Hospital of Changsha, Changsha City, Hunan Province 410035 China

**Keywords:** Older stroke survivors, Group-based ACT, Experiential avoidance, Protocol

## Abstract

**Introduction:**

Older stroke survivors usually experience various psychology disorders, such as post-stroke depression (PSD), which may be associated with high experiential avoidance (EA) and can seriously affect their quality of life. To date, the efficacy of group-based acceptance and commitment therapy (ACT) for older stroke survivors has not been established. The aim of this study is to investigate the effectiveness of group-based ACT on EA, PSD, psychological distress, and quality of life in older stroke survivors after group-based ACT.

**Methods and analysis:**

This study is a randomized, single-blind, wait-list controlled, parallel-arm trial. A total of 66 stroke survivors will be randomly assigned to wait-list control group or intervention group. Participants in wait-list control group will receive treatment as usual (TAU), while the intervention group will receive group-based ACT once a week for eight weeks. The primary outcome measure being EA, and the secondary outcome measures being PSD, psychological distress, and quality of life. Results of the two groups will be blindly assessed by professional evaluators at baseline (T0), post-treatment (T1), and one-month follow up (T2).

**Discussion:**

The results of this study will provide the first evidence for the effectiveness of a group-based ACT intervention in reducing EA, PSD, psychological stress, and improving quality of life for post-stroke survivors.

**Trial Registration:**

ChiCTR2200066361.

**Supplementary Information:**

The online version contains supplementary material available at 10.1186/s12906-023-04160-z.

## Strengths and limitations of this study

No study has explored the EA in older stroke survivors and related factors at present. Group-based ACT has been shown to have a positive impact on anxiety and depressive symptoms in stroke survivors, but no studies have explored the effect of group-based ACT on the psychological status in older stroke survivors.

Based on the previously published randomized controlled trials, this study used the group-based ACT to compare the effects of intervention on the psychological status of stroke survivors at different time points.

This study met the gold standard for clinical trial delivery and provided additional evidence for group-based ACT, which also helped to lay the foundation for the feasibility and effectiveness of group-based ACT for older stroke survivors.

The study had a short follow-up period.

In addition, its clinical nature prevents blinding of the participants and therapists.

**Supplementary Information:**

The online version contains supplementary material available at 10.1186/s12906-023-04160-z.

## Introduction

The global prevalence and incidence of stroke have increased significantly [1]. The prevalence of stroke increases with age, especially among the older patients [2]. According to the data obtained from 1,672 Chinese tertiary public hospitals, the average age of the 3,411,168 stroke cases during 2019 was 66 years old [3]. As treatment techniques continue to advance, more and more older stroke patients are expected to survive through active treatment. There are about 2.4 million new stroke patients and about 11 million stroke survivors in China every year [2, 3].

After surviving through the acute treatment, many older stroke survivors would be left with obvious complications such as cognitive, motor and sensory dysfunctions. They are often subject to partial or total loss of self-care ability, and are unable to play family and social roles, resulting in series of negative emotions [4–6]. Compared with stroke survivors in other age groups, older patients have poorer mobility, significantly reduced cognitive function and difficulty in regulating psychological distress. Therefore, subjectively, they may attempt to get rid of, avoid and escape from the previous painful experience of illness, and are unable to actively participate in social functional activities in accordance with their personal value goals, in a bid to restore their physical functions and return to normal life, which can be defined as experiential avoidance (EA) [7].

EA is essentially an avoidant coping process in which individuals associate certain cognitive and environmental cues with physiological and psychological discomfort and subsequently attempt to distance themselves behaviorally or psychologically from the discomfort [7]. Although it provides relief in the short term, it can sustain and often exacerbate psychological problems in the long term [8]. Studies have shown that the older persons are less likely to seek advice or to use strategies such as information seeking and emotional expression in the face of chronic illness, and tend to opt for fact-avoidance and behaviors that may stem from avoidance processes [9]. It is easy to imagine that within this context, painful emotional experiences would be suppressed or avoided altogether, and avoidance-based coping strategies in particular could become more prominent among older stroke survivors, which may lead to long-term mental health problems [10]. Therefore, EA may be an important predictor of mental health problems among the older stroke survivors, and may function as both an etiological and maintaining factor of post-stroke depression [10].

The most common neuropsychiatric outcome in stroke survivors is post-stroke depression (PSD) [11]. A meta-analysis of stroke survivors showed that PSD was the most common predictor of quality of life in stroke survivors [12]. Some studies suggest that PSD is longer, more severe and more difficult to treat [13], which may be related to the psychological rigidity and inflexibility of stroke survivors. Both the changes brought about by the disease and the psychological distress can significantly reduce the quality of life [5, 6, 14], thus increasing the risk of stroke recurrence and even death [15, 16]. With the transformation of the bio-psycho-social medical model, the rehabilitation of older stroke survivors has mainly focused on reducing the level of PSD and psychological distress, improving psychological resilience, and improving the quality of life. How to improve the physical and mental status of older stroke survivors, alleviate negative emotions, and improve their quality of life, has become an imperative problem to be solved by more and more scholars [17–19].

The Canadian Stroke Best Practice Recommendations, CSBPR) state that psychotherapy, including cognitive behavioral therapy, is one of the most commonly used strategies for post-stroke depression [16]. Acceptance and commitment therapy (ACT), referred to as the third wave of CBT, is a kind of behavioral therapy based on functional contextualism and relational framing theory [20]. The core component and the ultimate goal of ACT theory is to improve the individual’s psychological flexibility [21], that is, to fully feel, perceive and accept the things and emotions you experience at the moment, rather than focusing on avoiding painful memories or negative emotions in reality, and to make behavioral changes according to the direction of their own values, so as to change the relationship between cognition and emotion. ACT has been increasingly used in the psychological aspect of stroke survivors [5, 22, 23]. It can effectively relieve the psychological distress of stroke survivors and improve their quality of life, which may be related to ACT’s ability to reduce patients’ EA and improve their psychological flexibility.

In groups, people can socialize with peers with similar symptoms, have the opportunity to improve one’s altruism and empathy, and feel useful by helping others. In addition, group therapy results in a more cost-effective professional/client ratio [24]. In particular, group therapy provides an opportunity for depressed older adults, who often live in isolation and have lost significant relationships, capacities, or occupations, to once again feel connected with and useful to the society, as well as to (re)discover their meaning in life. Therefore, group-based ACT may have more unique therapeutic benefits, such as reducing feelings of isolation, providing opportunities to learn from the experience of other attendees, and developing new coping strategies and behavioral patterns based on such shared learning for older stroke survivors in China.

A study by Niu et al. [23] showed that group-based ACT could effectively relieve depressive symptoms and improve neurological function in PSD, thereby improving the quality of life of patients. One research supported the applicability of the ACT core processes in a variety of clinical populations, including older people [25]. Although relevant psychological studies have been carried out in the older population, and there are studies of group-based ACT for post-stroke depression, the application effect of group-based ACT in Chinese older stroke survivors needs to be further explored. Whether group-based ACT can be used as a supplementary study of TAU to enhance the psychological health of older stroke survivors has not been confirmed. This study aims to investigate the effectiveness and applicability of the group-based ACT intervention for older stroke survivors based on previous studies.

Therefore, we propose the following hypotheses: (1) ACT is an effective cognitive and behavioral intervention to improve EA, PSD, psychological distress, and quality of life of older stroke patients; and (2) compared with TAU alone, the group-based ACT may have better results on the psychological status of the older stroke survivors.

## Methods and analysis

### Patient and public involvement

No patient involved.

### Study design

**Fig. 1:**
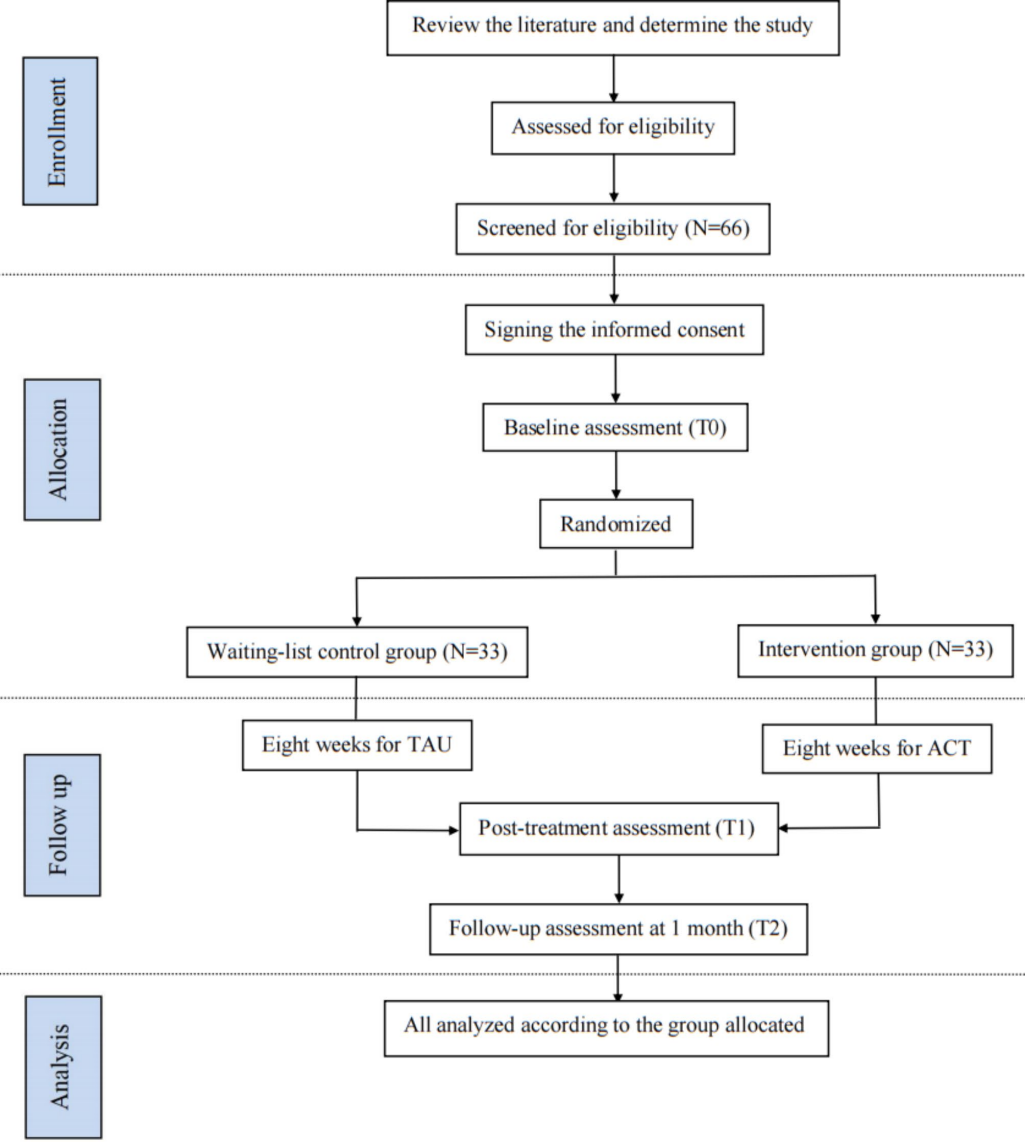
Flow diagram of the study

This study is a randomized, single-blind, wait-list controlled, parallel-arm trial. Older stroke survivors will be selected from a tertiary hospital in China using convenience sampling between December 2022 to March 2023. Older stroke survivors who meet the inclusion criteria will be informed of the purpose and methods of the study and sign the informed consent forms through face-to-face communication. Then, they will be randomly assigned to either a wait-list control group (TAU group) or an intervention group (group-based ACT group). EA, PSD, psychological distress and quality of life of patients will be blindly evaluated at baseline (T0), post-treatment (T1), one-month follow up (T2) for both groups (see Fig. 1).

Through literature review, qualitative interviews and group discussions, this study established an initial intervention program of group-based ACT for older stroke survivors. Two rounds of Delphi expert consultations were conducted on the group-based ACT program via email for initial intervention program. The recovery rates of expert questionnaires in the two rounds were 70% and 100%, respectively, with Ca standing at 0.95, Cs at 0.87 and Cr at 0.91, indicating that the authority of experts was high and the reliability of consultation results was effectively guaranteed. The Kendall’s W coefficients of the two rounds were 0.130 and 0.126 respectively (P < 0.05), and the experts’ opinions tended to be consistent.

According to the expert opinions and the characteristics of elderly stroke, the intervention program was adjusted as follows: older stroke patients were prone to coughing and choking when eating raisins in the traditional mindful diet, so the program was adjusted to sour date cake or chocolate; In addition, in the intervention program, other members of the group-based ACT intervention team would assist in completing the game, considering if the older population had physical impairment.

### Eligibility criteria

Inclusion criteria are: (1) conforming to the World Health Organization diagnostic criteria of stroke and confirmed by magnetic resonance imaging (MRI) or computed tomography (CT); (2) age ≥ 60 years old; (3) disease duration of more than half a year and having passed the acute stage and standard treatment [26]; (4) reporting a clinically significant level of psychological distress (Hamilton Depression Scale scored ≥ 8); (5) having the ability to communicate on a daily basis; (6) agreeing to participate and provide informed consent; and (7) having the ability to use WeChat, which is a multi-purpose social media, messaging and payment app developed in China.

Exclusion criteria are: (1) having other organic encephalopathy such as respiratory, cardiovascular, digestive and other serious physiological diseases; (2) having a history of alcohol or other psychoactive substance abuse or dependence; (3) having dementia, a recognized complication of stroke; (4) limb function weakened, and could not tolerate sitting for one hour.

### Sample size calculation

In this study, the sample size was calculated using PASS 16.0 Software. The sample size estimation formula of the mean of the two groups is adopted for calculation: The sample size ratio of the two groups was 1:1, that is, the Group Allocation was Equal(N1 = N2); Refer to Xiuli et al.s’ study [27], The control group and intervention group of HAMD scores, respectively (8.9 ± 4.0) and (6.5 ± 2.9). The α is the type I error probability, i.e. the test level or significance level, which is 0.05 on both sides in this study, the confidence level is 95%, ${u}_{\alpha }$ is 1.96. The β is class II error, which is 0.1, ${u}_{\beta }$ is 1.28.

$${n}_{1}={n}_{2}=2{\left[\frac{\left({u}_{\alpha }+{u}_{\beta }\right)}{\delta /\sigma }\right]}^{2}+\frac{1}{4}{{u}_{\alpha }}^{2}$$

According to the above formula, the sample size of each group was about 25 cases. Taking into account the sample loss and other problems, each group was expanded by 30%. Therefore, the final sample size for each group was approximately 33 cases, a total of 66 cases were required.

### Randomization

Randomization is performed by an independent researcher. Patients who volunteered for the study were numbered in the order of their admission. Sixty-six random numbers were generated using IBM SPSS Statistics for Windows (Version 26.0 Armonk, NY: IBM Corp) in descending order. Digits 1st through 33rd of the random-number sequence were numbered as the wait-list control group, and the remaining digits 34th through 66th were numbered as the intervention group.

### Interventions

#### Wait-list control group

The wait-list control group will receive TAU treatment for eight weeks, and the intervention will take place in a separate room in the inpatient building of the supporting unit, away from the ACT intervention site. When the intervention group has completed all the ACT procedures, the wait-list control group will receive the same ACT intervention as the intervention group. The TAU intervention is as follows. (1) Disease knowledge guidance, including introducing to patients the causes of stroke, how to identify stroke, clinical manifestations, treatment methods and other knowledge, so that patients can have a more comprehensive understanding of stroke; (2) Medication guidance and health education, including the mode of medication, time of medication, precautions for medication, possible side effects after medication and how to deal with them; (3) Basic psychological guidance, including counseling, persuasion and other forms, enabling patients to feel respect and care, increase their trust in medical staff, and rebuild their confidence to resume normal life; (4) Rehabilitation guidance, including rehabilitation training timing, rehabilitation training methods and precautions; (5) Psychological nursing, including encouraging patients, timely answering their doubts in the process of rehabilitation treatment, reducing their fear of disease, and establishing their confidence in early recovery.

### Intervention group

All interventions received by the intervention group were carried out in a separate activity room with an area of 20 m^3^ to ensure no interference. The activity room will be equipped with seats approximately, drinking water, and other necessary items. Moreover, necessary first-aid items will be also provided to ensure patient safety. The research team will provide materials and related things for ACT. Throughout the intervention, the health care workers will keep the patient safe at all times.

For the intervention group, group-based acceptance and commitment therapy intervention will be given for 8 weeks in addition to TAU, which will be completed by 2 graduate students, both of whom were systematically trained group-based ACT consultants, and will also lead the group consultation activities. Each group-based ACT intervention will consist of 10 ~ 12 people, once a week for 40 to 60 min, for 8 consecutive weeks. The content consists of eight sessions: confronting the agenda, accepting everything, objectively observing cognition, the hours, feeling yourself, identifying your value, looking forward, and review. The specific intervention content of each module is shown in Table 1.

**Table 1:** An overview of the group-based ACT program for older stroke survivors

Session	Content
Confronting the Agenda	Start with an “Ice-breaking tour” (5–10 min) Patient self-introduction (10–15 min) Introduction of ACT team members and content (5 min) Mindfulness exercises (10–15 min) Home task (5 min)
Accepting everything (Acceptance)	Begin with a mindfulness exercise and homework review (5–10 min); Watch a video of sinking into a swamp (10-15 min); Play the game “Chinese Finger Glove” (10–15 min); Share and summarize (10–15 min); Home task (5 min).
Observing cognition objectively (Cognitive Defusion)	Homework review (5–10 min); Play the video “Passengers on the Bus” (5–10 min); Play the game “Folder push” (10 to 15 min); Share and summarize (10–15 min); Home task (5 min).
The Hours (Being Present)	Begin with a review of last week’s homework (5-10 min); Mindful eating (10 min); Mindful breathing exercise (10-15 min); Share and summarize this activity (5–10 min); Home task (5 min).
Feeling yourself (Self as Context)	Begin with a mindfulness exercise and homework review (5-10 min); Play the video “Sky and Weather” (5 min); Share and summarize (15-20 min); Share poems, stories or elements of Chinese culture with the patients (5–10 min); Home task (5 min).
Identifying your value (Values)	Start with a few people sharing thoughts on last week’s homework (5–10 min); Play the game “Life Button” (15-20 min); Share and summarize (5-10 min); Home task (5 min).
Looking forward (Committed Action)	Start with a few people sharing thoughts on last week’s homework(10-15 min); Set goals (20–25 min); Share and summarize (5–10 min); Home task (5 min).
Review	Start with homework review (5 min); Play photos and videos (5–10 min); Share feelings (15-20 min); Give blessings, encouragement, and gifts (10–15 min).

### Blinding

The researcher conducting baseline assessments (T0) will be blinded to participant treatment allocation, as participants will also be blinded to randomization after the baseline assessment (T0). It is not possible for the treating psychologists providing the intervention to be blinded. However, an independent assessor who will join to complete the post-treatment (T1) and one-month follow up assessment (T2), will be blinded to the condition. The success of blinding will be assessed by having independent assessors (i) indicate which condition they thought the participant will be randomly assigned to and (ii) indicate whether the participant will disclose their treatment condition or not. Data analysis will be undertaken by a graduate student with no direct contact with the individual research group to remain blinded to the treatment conditions. It is expected that unblinding will be not needed for independent assessors or statisticians, therefore in this study, unblinding is not permissible.

### Quality Control

- The therapist will post reminders and trailers in the WeChat group before each intervention in ACT group.
- The therapist will summarize and comment on the day after each session.
- The therapist will clock in the WeChat group to supervise the completion of the homework of the patients, and provide comments and feedback to the patients who actively share the experience after the completion of the homework. Patients can also communicate with each other in the WeChat group.
- Considering the declining vision and cognitive ability of the older population, large-sized intervention explanatory sheets will be distributed in this study, and each patient will be provided with a helper to understand the intervention content.
- If required, the measures can be read out to the participant by the assessor to assist with understanding, and a separate sheet with the measure’s Likert response scales will be provided to the participant in a larger font to compensate for visual, attention/cognitive, and reading impairments in the older adults.
- We will compensate for the time and effort the study participants put into the study. In the wait-list control group, we will help them understand the disease knowledge, distribute the recommended recipe list of stroke survivors, the prevention manual of recurrent stroke, answer questions at any time and so on. The intervention group will receive these equal compensations, and all the patients will be encouraged to complete the intervention by compensating them with daily necessities, such as tissues, masks, and swabs, before each week of the intervention. Of course, the wait-list control group will also have equal compensation when the formal intervention ended.

### Statistical analysis

The statistical analysis of this study can be divided into four parts. Firstly, the IBM SPSS Statistics for Windows (Version 26.0 Armonk, NY: IBM Corp) would be used in this study. Data processing was performed to review missing data, followed by screening for normality for all outcome measures and for each treatment group. Secondly, inter-group comparisons were performed prior to the main analysis, and differences between baseline groups were analyzed using Chi-square test and independent sample T-test to verify the success of randomization. If the relevant data did not conform to a normal distribution, the generalized estimation equation (GEE) would be used for analysis. In addition, a preliminary analysis would be performed on an intention-to-treat basis using Generalized linear mixed model (GLMM) to investigate the differential effects of each treatment condition. For the post-treatment analysis in the three treatment conditions, the analysis will focus on the linear time effect, treatment conditions and interaction. Finally, a linear mixed model repeated measures (MMRM) was used to study inter-group differences with primary and secondary outcome measures as dependent variables. The model would include processing conditions and time as classification variables, as well as their interaction effects. The baseline adjusted mean difference between groups at each time point would be calculated using a 95% confidence interval, with the value of alpha set at 0.05. The analysis would focus on the estimated mean differences between primary and secondary outcome indicators at different time points.

### Primary outcome measures

#### The Acceptance and Action Questionnaire-II, AAQ-II

AAQ-II scale, a seven-item questionnaire developed by Bond et al. [28] in 2011, was used to assess EA. In the questionnaire, items are scored on a 7-point Likert scale with a total score ranging from 0 to 49, and a higher score indicating more EA. The internal consistency of the AAQ-II is good (Cronbach α = 0.84) [28]. In 2013, Yao et al. [29] measured the reliability of Chinese version of Acceptance and Action Questionnaire-II (AAQ-II), which consists of 10 items rated on a Likert scale of 1 ~ 7 points. The score for items 2 ~ 5 and 7 ~ 9 is reversed. The total possible score ranges from 10 to 70, with higher scores representing higher levels of general acceptance. The coefficient of Cronbach’s α for the Chinese version was 0.705, and the standardized item alpha was 0.758, indicating a good internal consistency of the questionnaire.

### Secondary outcome measures

#### The hamilton depression scale, HAM-D

The HAM-D scale, developed by Hamilton [30] in 1960, is the most commonly used scale to assess post-stroke depression. The revised 24 Chinese HAMD items is also used in this study. A total of 24 items are divided into 7 dimensions: anxiety/somatization (6 items), body quality (1 item), cognitive impairment (6 items), day-night changes (1 item), sluggishness (4 items), sleep disorders (3 items), and feelings of hopelessness (3 items). The scale was scored using the likert 5 rating method (no = 0, mild = 1 min, moderate = 2 points, and severe = 3 points, heavy = 4). The total score of the scale ranged from 0 to 76, with > 35 being classified as severe depression, 21–35 as mild to moderate depression, 8–20 as possible depression, and < 8 as no depression [31]. The Cronbach’s α coefficient of the Chinese version of the HAMD-6 was 0.91, and the intra-group correlation coefficient (ICC) for retest reliability was 0.81 [32].

### Distress thermometer, DT

Developed by Gilson [33], DT could reflect the patient’s psychological distress level during the past week. The scale consists of two parts: (1) DT, a scale of 0 ~ 10 points (0: no pain, 10: extreme pain), with higher scores representing more severe psychological distress of the patient. (2) The 39-question list is a list of questions related to psychological pain examined by the patients, with a total of 6 dimensions and 39 items. In this study, only the psychological pain thermometer score was used and it was divided into three levels: 0 ~ 3 for mild; 4 ~ 6 for moderate, and 7 ~ 10 for severe. The Cronbach’s α coefficient of this scale was 0.808, which has good reliability and validity.

### Stroke-specific quality of Life, SS-QOL

SS-QOL, developed by Williams et al. [34], is used to assess the quality of life. It is a reliable and effective tool for measuring health-related quality of life in Chinese stroke survivors [35]. The scale consists of 11 domains (47 items), including activities, mood, energy and family roles, language, upper extremity function, relationships, thinking, vision, basic needs, personality, leisure and work, and transfer, each with a score of 1 ~ 5 points. The internal consistency of SSQL-C is high. Cronbach’s alpha for the total scale was 0.93, and Cronbach’s alpha for all 11 domains ranged from 0.63 to 0.90 [35].

## Discussion

An increasing number of randomized controlled trials have been conducted to study the effectiveness of group-based ACT in treating psychological status, and the corresponding curative conclusions have been obtained. Among them, the subjects mainly included nurses [36], adolescents with multiple functional somatic syndromes [37], and mothers of children with autism spectrum [38], older veterans [39] and so on. But until now, there have been no studies applying the group-based ACT to older stroke patients. In terms of psychological intervention for stroke survivors, Euan et al. [40] developed a novel peer support intervention to promote resilience after stroke. Majumdar et al. [41] conducted group-based ACT in stroke survivors and found that this approach significantly reduced depression and improved psychological health status in stroke survivors. However, its efficacy in older stroke survivors is unknown and needs further discussion.

This is the first randomized controlled trial to test the efficacy of group-based ACT as a complementary therapy to TAU in improving EA, PSD, psychological distress, and quality of life in older stroke survivors, thereby facilitating rehabilitation and providing evidence-based support for psychological remodeling in older stroke survivors. Through group psychological intervention, older stroke survivors could share the experience of life-changing and psychological growth caused by disease, communicate their inner thoughts and feelings with patients and therapists, and may promote their self-acceptance.

Older stroke survivors may have lower cognitive and emotional regulation abilities than younger patients or patients with other diseases, and may have more EA in response to past painful experiences and acute treatment. As previously mentioned, the core purpose of group-based ACT is to improve patients’ EA and thus their psychological distress and quality of life. Therefore, EA will be used as the primary outcome indicator in this study for the following reasons. First, the reduction of symptoms (such as psychological distress) is largely considered to be a beneficial therapeutic consequence of EA. Second, as the mental flexibility of older stroke survivors improves, their psychological stress also tends to decrease [18]. We estimated that improvements in psychological distress, PSD, and quality of life were caused by the increase in EA. Finally, the selection of EA as the primary outcome measure will help to innovate the comparability of TAU complementary therapy studies.

It is a new strategy for stroke survivors to reduce EA, PSD and psychological distress, and improve quality of life through group-based ACT intervention. This work has the potential to positively impact psychological interventions for post-stroke survivors and increase our understanding of how and for whom these interventions are most effective.

### Ethical considerations and dissemination

Throughout the study, ethical requirements will be strictly observed at all stages. We will maintain the right to information of the research objects, abide by the principle of confidentiality, and formulate relevant risk avoidance and disposal plans.

- Follow the principle of informed consent. We will fully explain to the participants information about the purpose, content, potential risks, direct or indirect benefits and other information of the research to ensure that they understand and voluntarily participate in the study and sign informed consent forms.
- Follow the principle of confidentiality. All personal information obtained during the research will be kept confidential and used for research purposes only. Upon completion of the research, the research materials will be destroyed immediately. All publications and papers based on the data from this study will not disclose any identifiable personal information about the subject.
- Risk avoidance and disposal plan. (i)In the process of data collection and intervention, researchers will give detailed explanations, personal demonstrations and guidance to measurement and exercise-related movements, and teach participants to do the relevant movements safely to avoid unnecessary injuries. At the same time, rest is arranged as appropriate by observing and inquiring about participants’ status. (ii)Participants are informed that in case of adverse events, caregivers and project staff should be notified immediately, and project staff should assist in contacting the relevant medical units for treatment, and report to the ethics Committee. (iii)In the whole process of on-site intervention, the medical staff will ensure safety and avoid accidents.
- Follow the principle of equal treatment in the control group. After the intervention, if the intervention proves to be effective, the same intervention and content instruction will be given to the wait-list control group.

### Electronic supplementary material

Below is the link to the electronic supplementary material.

**Supplementary Material 1: Table S1**. ACT metaphor and their content

## Data Availability

Not applicable.
