# Supplementary material for: Effect of group-based acceptance and commitment therapy on older stroke survivors: study protocol for a randomized controlled trial
Source: BMC Complement Med Ther. 2023 Oct 6;23:353. doi: 10.1186/s12906-023-04160-z (PMC10557283; doi:10.1186/s12906-023-04160-z)
Supplement: Supplementary file 1 — Supplementary Material 1: Table S1. ACT metaphor and their content [file 12906_2023_4160_MOESM1_ESM.docx]

**Supplementary file**

**Table S1. ACT metaphor and their content**

| ACT metaphor | Content |
| --- | --- |
| Chinese Finger Glove | A Chinese finger cuff is a small, tubular, elastic toy. The method of the game as follows: first, the left and right hands each stick a finger firmly into the finger cuff, and then, try to pull out the two fingers without breaking the finger cuff. People's first reaction is usually to try to pull the finger out, but the result is to make the finger cuff more firmly on the tip of the finger, the finger cuff does not loosen, but makes the ring buckle more stronger. |
| Passengers on the Bus | Imagine life is like a journey, and you’re the driver of your bus. Over the course of your life, various passengers have boarded your bus. They reflect your thoughts, feelings, and all kinds of inner states. And then there are passengers that you wish had not boarded the bus; they can be ugly, scary, and nasty. You may try to avoid them, distract yourself, or throw them off the bus, but they are your inner states, so you can’t get rid of them. However, while the bus is stopped, you’re not moving in the direction that’s important to you. By fighting and struggling with the passengers or giving in to them, you, the driver, are not in control of your journey of life, and it’s likely that you are not heading in a direction that is important to you. (https://www.youtube.com/watch?v=vGGAI60U0uI). |
| Folder push | The participants were provided with a folder, and they covered their face with their hands and kept it as close to their face as possible, so that they could neither see outside nor show their world to others. Not only did they lose contact with the world, but they might not be able to live a normal life, such as hugging others with their hands. Then bring it down from your face and learn to hold it in your lap, so you can do anything useful without any restraint, as opposed to covering your face with a folder or pushing the folder. |
| Sky and Weather | Your observing self is like the sky. Thoughts and feelings are like the weather. The weather changes continually, but no matter how bad it gets, the weather cannot harm the sky in any way. The mightiest thunderstorm, the most turbulent hurricane, the most severe winter blizzard–these things cannot hurt or harm the sky. |
| Life Button | Different buttons symbolize different things, such as wealth, health, beauty and so on. By discarding other buttons and keeping the most important ones for yourself, you can help yourself to clarify your value and what you are pursuing, and help you set your life goals. |
